# Phthisis

**Published:** 1876-04

**Authors:** 


PHTHISIS—Its Morbid Anatomy, Etiology, Symptomatic Events and Complications, Fatality and Prognosis, Treatment and Physical Studies, in a series of clinical studies. By Austin Flint, M.D., Professor of Principles and Practice of Medicine in Bellevue Hospital College, Fellow of New York Academy of Medicine, Associate Fellow of the College of Physicians of Philadelphia, Honorary member of Clinical Society of London, etc. Philadelphia: Henry C. Lea.

This work consists in the careful recording of nearly 700 cases of phthisis, and in abstracting, grouping, and analysing them with reference to practical deductions. It is divided into six chapters: the 1st, treats of morbid anatomy; 2d, of etiology; 3d, symptomatic events and complications; 4th, fatality and prognosis; 5th, treatment; 6th, physical signs and prognosis.

The eminent position occupied by the author in the medical world, both as a writer and teacher of medicine, should commend this work to the profession at large. It is a work of 446 pages, and the truths contained therein shine out like "apples of gold in pictures of silver."

